# In Vitro Evidence for Oral Soft Tissue Regenerative Effects of a Polynucleotide–Hyaluronan Formulation

**DOI:** 10.1002/cre2.70253

**Published:** 2025-11-18

**Authors:** Ina Mladenova, Xiaoqing Song, Cristina Nica, Anton Sculean, Maria B. Asparuhova

**Affiliations:** ^1^ Laboratory of Oral Cell Biology, Department of Periodontology, School of Dental Medicine University of Bern Bern Switzerland

**Keywords:** connective tissue grafts, growth factors, hyaluronic acid, oral soft tissue wound healing, polynucleotides

## Abstract

**Objectives:**

We aimed to investigate the effects of a polynucleotide–hyaluronan (PN–HA) formulation on human palatal fibroblasts (HPFs) and oral epithelial cells (OKF6/TERT‐2) to assess its potential application in oral soft tissue reconstructive procedures.

**Material and Methods:**

The impact of PN–HA on cell viability, migration, and proliferation was assessed using functional assays. Gene expression of wound healing‐related growth factors, inflammatory cytokines, and keratinization markers was evaluated by qRT‐PCR, while protein expression and kinase signaling were analyzed via immunofluorescence and immunoblotting.

**Results:**

PN–HA preserved cell viability and significantly (*p* < 0.05) enhanced migration of both HPFs and OKF6/TERT‐2 compared to untreated control cells. While it significantly (*p* < 0.01) increased proliferation in HPFs, no direct proliferative effect was observed in OKF6/TERT‐2 monocultures. In HPFs, PN–HA upregulated TGFB1, PDGFB, and FGF2 genes encoding the respective pro‐migratory/pro‐proliferative growth factors, whereas TGFB3 and EGF were selectively induced in OKF6/TERT‐2. PN–HA markedly increased pro‐inflammatory cytokine expression and activated p38 MAPK signaling in epithelial cells, alongside ERK1/2 activation and moderate increase in adenosine A2A receptor expression in both cell types. A pronounced upregulation of keratinocyte differentiation markers was observed at both mRNA and protein levels. Notably, in epithelial‐fibroblast cocultures, PN–HA significantly (*p* < 0.01) enhanced epithelial proliferation, likely mediated by increased FGF7 and HGF expression in fibroblasts.

**Conclusions:**

PN–HA promotes oral fibroblast migration and proliferation, induces epithelial differentiation, and stimulates epithelial proliferation in coculture with fibroblasts. The observed cell‐ and context‐specific effects highlight the potential of PN–HA to support soft tissue regeneration and keratinization in periodontal reconstructive surgeries.

## 1 Introduction

Hyaluronic acid (HA), also known as hyaluronan, is an anionic, non‐sulfated glycosaminoglycan abundantly present in the extracellular matrix (ECM) and involved in maintaining its resilience (Bertl et al. 2015; Garantziotis and Savani 2019; Pilloni et al. 2025). HA is present in various body fluids, including saliva and gingival crevicular fluid (Embery et al. 2000; Pogrel et al. 2003) as well as in mineralized and non‐mineralized tissues, including the periodontium (Ohno et al. 2002). It plays a critical role in maintaining tissue hydration and modulating diverse cellular functions (Iaconisi et al. 2023). Our previous research demonstrated that native and crosslinked HA formulations are biocompatible and significantly enhance the proliferation, migration, and wound‐healing capacities of primary human oral fibroblasts (Asparuhova et al. 2019). Additionally, HA has been shown to stimulate the growth of osteoprogenitors and maintain their stemness, thereby playing a key role in regulating the balance between self‐renewal and differentiation during bone regeneration (Asparuhova et al. 2020). These in vitro results were corroborated by preclinical histological studies in canine models, which reported positive effects of HA on the periodontal wound healing in surgically created class III furcation (Shirakata et al. 2022), two‐wall intrabony (Shirakata et al. 2021), and gingival recession defects (Shirakata et al. 2021). Clinical studies further validated the efficacy of HA in treating single and multiple adjacent recession defects when used in conjunction with subepithelial connective tissue grafts (CTGs) and modified coronally advanced or laterally closed tunnel techniques (Guldener et al. 2020; Lanzrein et al. 2020; Pilloni et al. 2019, 2025; Rotundo et al. 2025). Its effectiveness in the treatment of intrabony periodontal defects has also reinforced its clinical relevance in regenerative periodontal surgery (Pilloni et al. 2021; Vela et al. 2024). Furthermore, repeated local delivery of HA as adjunctive treatment of residual pockets in periodontitis patients undergoing supportive periodontal care has resulted in fewer sites requiring re‐instrumentation at 3 months and higher pocket closure rate at 12 months (Bertl et al. 2024).

Similar to HA, polynucleotides (PNs) are natural polymers capable of binding water molecules and are ubiquitously distributed across tissues (Marques et al. 2025). They are released into the extracellular space by damaged or apoptotic cells (Rathbone et al. 1992). PNs influence various cellular activities through multiple mechanisms, including purinergic signaling via activation of the adenosine A2A receptor (ADORA2A) (Irrera et al. 2018; Marques et al. 2025) and the salvage pathway, which provides cells with supplementary nucleotides to support proliferation (Marques et al. 2025; Rathbone et al. 1992). PNs of animal origin, typically extracted from trout gonadal DNA, have been used in clinical practice for nearly two decades in various class III medical devices due to their wound‐healing properties (Colangelo et al. 2020, 2021; Lampridou et al. 2025). In an in vitro model of oral mucositis, PN demonstrated potential to modulate inflammation and improve wound healing (Picciolo et al. 2021).

Recent randomized clinical trials have shown that formulations combining HA and PN are effective in treating skin ulcers (Segreto et al. 2020), venous lower limb ulcers (De Caridi et al. 2016), and knee osteoarthritis (Stagni et al. 2021). Furthermore, a randomized split‐mouth clinical trial demonstrated that adjunctive use of a PN–HA‐based gel significantly reduced clinical signs of inflammation during subgingival re‐instrumentation of deep residual periodontal pockets (Pilloni et al. 2023). The combined application of PN and HA is expected to exert potent synergistic effects on the behavior of cells essential for wound healing. Formulations containing both compounds have been shown to stimulate the proliferation of gingival fibroblasts and enhance ECM deposition by upregulating collagen type I (Colangelo et al. 2021; Han et al. 2025) and proteoglycans (Han et al. 2025). However, detailed in vitro characterization of PN–HA formulations, essential for supporting their potential application in reconstructive periodontal surgery, is still lacking. Therefore, the aim of the present study is to investigate the in vitro effects of a commercially available PN–HA formulation on the metabolic activity, migration, proliferation, and differentiation of primary human oral fibroblasts and oral epithelial cells, which are key cellular players in oral soft tissue regeneration.

## 2 Materials and Methods

### 2.1 Cell Culture

Primary human palatal fibroblasts (HPFs) were isolated via the tissue explant technique (Asparuhova et al. 2019) from three systemically and periodontally healthy individuals below 30 years who had undergone periodontal surgery, for example, recession coverage using palatal subepithelial CTGs or crown lengthening, following signed informed consent and approval by the Ethics Committee, Bern, Switzerland (ethical code ID 2018‐00661 from 13.08.2018). In brief, each donor tissue sample was minced into 1 mm tissue explant pieces, which were then pipetted into a 25 cm^3^ tissue culture flask and cultivated in Dulbecco's modified Eagle's medium (DMEM; Thermo Fisher Scientific, Basel, Switzerland) supplemented with 10% fetal calf serum (FCS; Thermo Fisher Scientific) and 1% antimycotics/antibiotics (AA; Thermo Fisher Scientific). The oral epithelial cell line OKF6/TERT‐2 (Dickson et al. 2000), kindly provided by Prof. Dr. James Rheinwald (Boston, MS, USA), was maintained in complete keratinocyte serum‐free medium (KSFM; Thermo Fisher Scientific) supplemented with 0.2 ng/mL EGF (Peprotech, London, UK), 25 μg/mL bovine pituitary extract (Thermo Fisher Scientific), and 1% AA. Before treatment, HPFs (passages 1–5) and OKF6/TERT‐2 were starved for 24 h in 0.3% FCS/DMEM or basal KSFM (bKSFM; without EGF), respectively.

PN–HA (REGENFAST, Geistlich Pharma AG, Wolhusen, Switzerland) is a commercial formulation composed of two components: (1) PNs of natural origin at a final concentration of 10 mg/mL, and (2) hyaluronan (HA) of biotechnological origin, non‐crosslinked and with a molecular weight of approximately 1000–1300 kDa, also at a final concentration of 10 mg/mL. The PN–HA was applied throughout the study at a final concentration of 2.5 mg/mL based on concentration titration experiments (see cell viability assay), previous in vitro studies employing comparable concentrations of PN and HA (Han et al. 2025), and consistency with physiological HA concentrations (Gupta et al. 2019). In some cases, 1 × 10^5^ HPFs (lower chamber) and 5 × 10^4^ OKF6/TERT‐2 (insert) were cocultured using transwell inserts (Thermo Fisher Scientific) with 0.4 µm‐pore size, in the absence or presence of PN–HA diluted in bKSFM.

### 2.2 Cell Viability

Viability was measured using the CellTiter‐Blue assay (Promega, Madison, WI, USA) as described by Asparuhova et al. (2019). After 24 h of starvation, cells were resuspended in media containing PN–HA at the indicated concentrations and plated in triplicate at 5 × 10^3^ cells/well on 96‐well plates. Cells were cultured for 4 h before recording fluorescence intensity on Varioskan LUX (Thermo Fisher Scientific). Experimental values were normalized to the values of untreated cells (100% viability). Data represent means ± SD from three independent experiments performed with three different cell donors for the HPFs.

### 2.3 Cell Migration

Cell migration was assayed using transwell inserts (Corning, Amsterdam, The Netherlands) with 8 µm‐pore size as described by Asparuhova et al. (2019). After 24 h of starvation, 8 × 10^4^ HPFs or 5 × 10^4^ OKF6/TERT‐2 were plated in the top insert chamber in serum‐free‐DMEM or bKSFM, respectively. The lower chamber contained complete media with or without PN–HA. Cells were allowed to migrate across the filter for 22 h at 37°C before fixation and crystal violet‐staining. Images of triplicate inserts were acquired on an Olympus BX‐51 microscope. Migration was quantified by ImageJ, v1.54p. Data represent means ± SD from three independent experiments performed with three different cell donors for the HPFs.

### 2.4 Cell Proliferation

Proliferation rates of untreated and PN–HA‐treated mono‐ and co‐cultures of HPFs and OKF6/TERT‐2 were determined using a 5‐bromo‐20‐deoxyuridine (BrdU) incorporation assay (Roche, Basel, Switzerland) as described by Asparuhova et al. (2019). BrdU labeling was performed at 0, 24, 48, 72, and 96 h and its incorporation into newly synthesized DNA was determined according to manufacturer's instructions. Experimental values were normalized to the values of untreated cells at the time point 0. Data represent means ± SD from three independent experiments performed with three different cell donors for the HPFs.

### 2.5 qRT‐PCR

Quantitative RT‐PCR was used to investigate the expression of four groups of genes encoding (1) the proliferative markers MYBL2, BUB1, PLK1, MKI67, PCNA, CCNB1, CCND1, and CCNE1; (2) the growth factors TGFB1, TGFB3, PDGFB, FGF2, FGF7 (also known as KGF), HGF, and EGF; (3) the pro‐inflammatory factors IL1A, IL1B, IL6, and TNF; and (4) the keratinocyte differentiation markers KRT5, KRT14, KRT19, KRT1, KRT10, TGM1, IVL, FLG, and LORICRIN. Total RNA from untreated and PN–HA‐treated mono‐ and co‐cultures of HPFs and OKF6/TERT‐2 was isolated using the RNeasy Mini Kit (Qiagen, Basel, Switzerland). RNA was reverse‐transcribed and relative transcripts for the above genes, normalized to GAPDH, were measured using the primer sequences listed in Table S1 and FastStart Universal SYBR Green Master ROX (Applied Biosystems, Rotkreuz, Switzerland) on a QuantStudio 3 (Applied Biosystems). Data were analyzed using the ∆∆Ct method. Samples were run in duplicates. Data represent means ± SD from three independent experiments performed with three different cell donors for the HPFs.

### 2.6 Immunoblotting and Immunofluorescence

Whole‐cell lysates from untreated or PN–HA‐treated HPFs and OKF6/TERT‐2 were prepared in RIPA buffer, run on 10% SDS–PAGE, and transferred to nitrocellulose membranes (Sigma, Basel, Switzerland) as described by Asparuhova et al. (2019). Proteins of interest were visualized using anti‐ADORA2A (Thermo Fisher Scientific), anti‐phospho‐Erk1/2 (Cell Signaling Technology, Danvers, MA, USA), anti‐phospho‐p38 (Cell Signaling Technology), and anti‐vinculin (Sigma) antibodies followed by horseradish peroxidase (HRP)‐conjugated secondary antibodies (MP Biomedicals, Santa Ana, CA, USA) for detection with chemiluminescence HRP‐substrate (Thermo Fisher Scientific). The expression of ADORA2A or the phospho‐proteins was quantified relative to the expression of the loading control or respective total proteins by densitometry using ImageJ, v1.54p. Data represent means ± SD from three independent experiments performed with three different cell donors for the HPFs.

Immunofluorescent staining of untreated and PN–HA‐treated cells was performed as described by Asparuhova et al. (2011) using anti‐vimentin (Thermo Fisher Scientific), anti‐E‐cadherin (Proteintech, Manchester, UK), anti‐keratinocyte transglutaminase (Proteintech), anti‐involucrin (Thermo Fisher Scientific), anti‐filaggrin (Thermo Fisher Scientific), and anti‐loricrin (Thermo Fisher Scientific) antibodies. F‐actin was labeled with phalloidin‐TRITC (Sigma) that was applied simultaneously with Alexa Fluor 488 (Thermo Fisher Scientific) secondary antibody. Nuclei were labeled with 4′,6‐diamidino‐2‐phenylindole (DAPI; Sigma). Images were acquired using an Olympus BX‐51 (Olympus Life Sciences Solution, Tokyo, Japan) equipped with the fluorescent filters U‐MWIBA3 for Alexa Fluor 488, U‐MWIGA3 for TRITC, and U‐MNUA2 for DAPI.

### 2.7 Statistical Analysis

Statistical analyses were performed using GraphPad InStat Software, v3.05. Data from independent biological replicates were averaged from technical repeats to ensure statistical independence. Data distributions and variance homogeneity were inspected and found suitable for parametric analysis. Differences between two groups were assessed by Student's *t*‐test and between multiple groups by one‐way analysis of variance with Tukey's post hoc test. Values of *p* < 0.05 were considered statistically significant.

## 3 Results

### 3.1 PN–HA Does Not Impair Viability or Morphology of Oral Fibroblasts and Epithelial Cells

**Figure 1:**
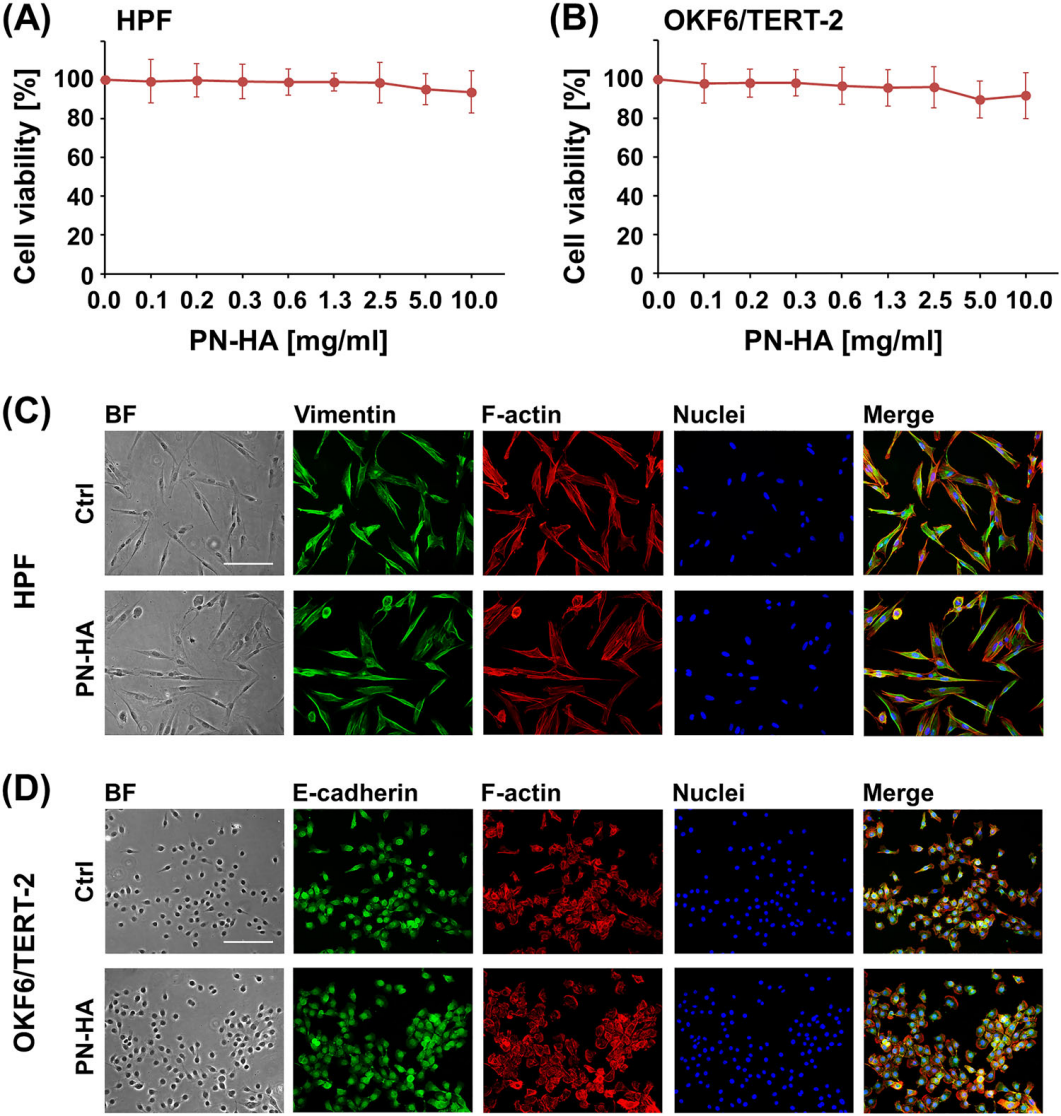
PN–HA maintains viability and morphology of oral fibroblasts and epithelial cells. (A, B) Viability of HPFs and OKF6/TERT‐2 treated with increasing concentrations of PN–HA (0–10 mg/mL) was assessed using the CellTiter‐Blue assay. Experimental values are expressed relative to untreated controls (0 mg/mL, set to 100%). Data represent means ± SD from three independent experiments performed with three different cell donors for the HPFs. (C, D) HPFs and OKF6/TERT‐2 cells were treated with 2.5 mg/mL PN–HA or left untreated (Ctrl) for 24 h, followed by immunofluorescent staining. Vimentin (green, HPFs), E‐cadherin (green, OKF6/TERT‐2), F‐actin (red), and nuclei (blue) were visualized on fixed and permeabilized cells. Scale bar, 500 μm. BF, bright field.

Both HPFs and OKF6/TERT‐2 cells retained high viability after exposure to PN–HA across a concentration range of 0.02–2.5 mg/mL, with viability ≥ 98.5% for HPFs (Figure 1A) and ≥ 95.9% for OKF6/TERT‐2 cells (Figure 1B), relative to untreated controls (100%). A mild, nonsignificant reduction in viability (4%–6%) was observed at 5.0 and 10.0 mg/mL.

Morphological analysis after 24 h treatment with 2.5 mg/mL PN–HA showed preserved cellular architecture in both cell types, as confirmed by vimentin staining in HPFs (Figure 1C) and E‐cadherin staining in OKF6/TERT‐2 (Figure 1D). PN–HA‐treated OKF6/TERT‐2 cells also exhibited early clustering behavior, suggestive of epithelial differentiation.

### 3.2 PN–HA Strongly Increases the Migratory Capabilities of Oral Fibroblasts and Epithelial Cells but Significantly Stimulates Proliferation of Oral Fibroblasts Only

**Figure 2:**
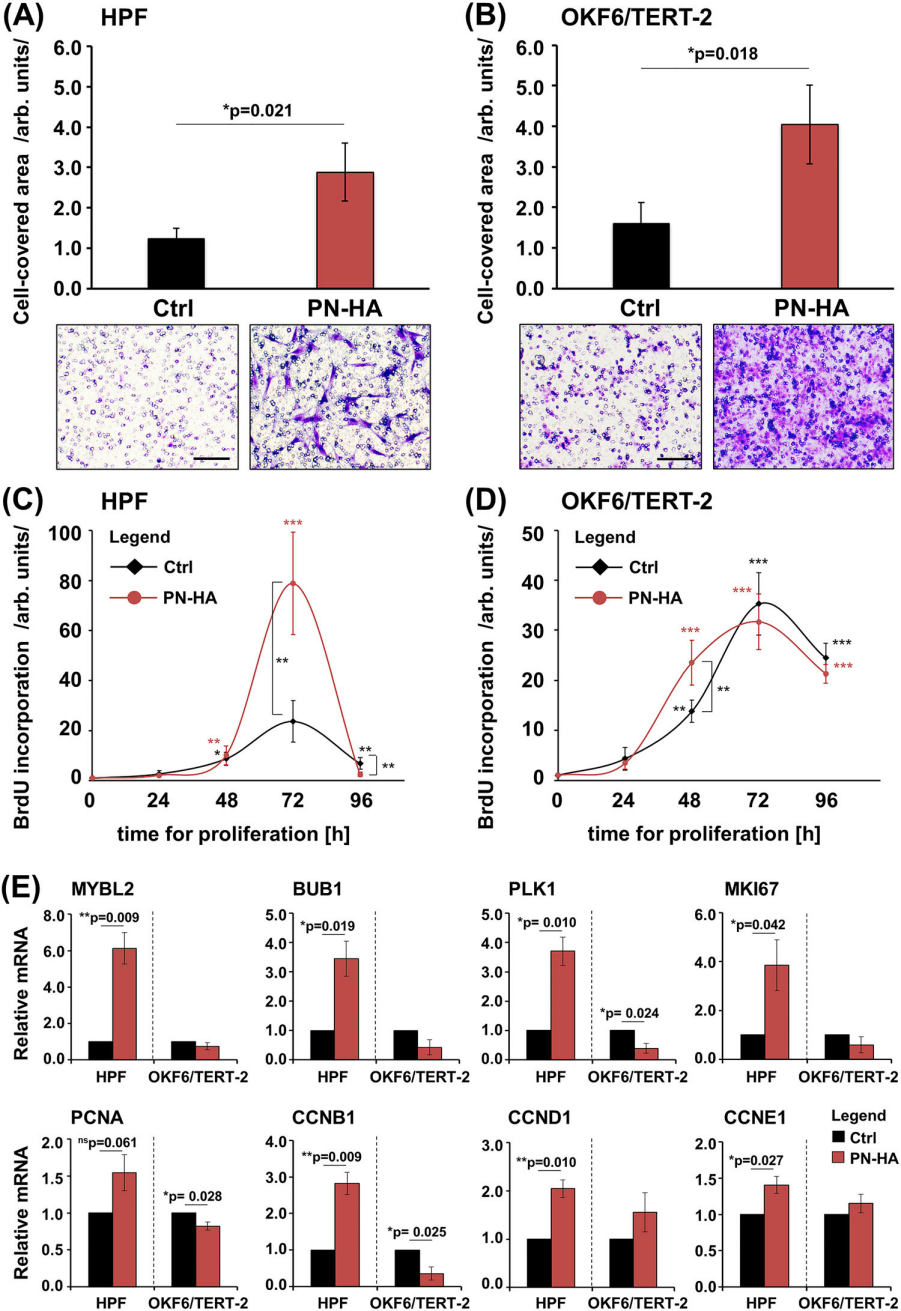
PN–HA strongly increases the migratory capabilities of oral fibroblasts and epithelial cells but significantly stimulates proliferation of oral fibroblasts only. (A, B) Migration of HPFs and OKF6/TERT‐2 cells towards PN–HA was evaluated by transwell migration assay using 8 μm pore‐size filters. Cell migration was quantified by measuring the area covered by cells on the underside of the filter. Data represent means ± SD from three independent experiments performed with three different cell donors for the HPFs. Significant differences to control (Ctrl) cells, *p < 0.05. Representative images of the staining in each of the experimental groups are shown below the graphs. Scale bar, 500 μm. (C, D) Proliferation rates of PN–HA‐treated HPFs and OKF6/TERT‐2 cells were assessed by BrdU incorporation assay immediately after plating (0 h) and at 24, 48, 72, and 96 h. Data represent means ± SD from three independent experiments performed with three different cell donors for the HPFs. Significant differences to control (Ctrl) cells at the time point 0 unless otherwise indicated, ***p < 0.001, **p < 0.01, and *p < 0.05. (E) Effect of PN‐HA on MYBL2, BUB1, PLK1, MKI67, PCNA, CCNB1, CCND1, and CCNE1 mRNA levels in HPF and OKF6/TERT‐2. Cells were treated with PN–HA for 72 h before total RNA was extracted and analyzed by qRT‐PCR. Values normalized to GAPDH are expressed relative to the values of control (Ctrl) cells. Data represent means ± SD from three independent experiments performed with three different cell donors for the HPFs. Significant differences to the respective control, **p < 0.01 and *p < 0.05.

Next, we investigated the migratory capacity of HPFs and OKF6/TERT‐2 cells towards PN–HA using a transwell assay. Compared to untreated controls, PN–HA significantly increased migration in both HPFs and OKF6/TERT‐2 by 2.3‐ and 2.5‐fold, respectively (*p* < 0.05; Figure 2A,B).

We next assessed the proliferative response to PN–HA using BrdU incorporation assay. PN–HA‐treated HPFs showed a marked increase in DNA synthesis, reaching a 3.3‐fold higher proliferation rate compared to control at 72 h (*p* < 0.01; Figure 2C). While OKF6/TERT‐2 cells also showed a significant increase in proliferation at 48 h (1.7‐fold, *p* < 0.01), this effect plateaued thereafter, resulting in overall proliferation rates comparable to the ones of control cells (Figure 2D). These observations were further supported by qRT‐PCR analysis of cell cycle‐related genes. In HPFs, PN–HA significantly upregulated the majority of tested proliferation markers at 72 h, namely MYBL2, BUB1, PLK1, MKI67, CCNB1, CCND1, and CCNE1, with the exception of PCNA, which remained unchanged (Figure 2E). In contrast, PN–HA‐treated OKF6/TERT‐2 cells showed a slight but significant downregulation of PLK1, PCNA, and CCNB1, while the expression of the remaining markers remained unaffected.

Collectively, these data indicate that PN–HA robustly enhances the migratory capacity of both oral fibroblasts and epithelial cells, while significantly promoting proliferation in fibroblasts only.

### 3.3 Distinct Growth Factors and Cytokines Are Modulated by PN–HA in Oral Fibroblasts and Epithelial Cells, Alongside Cell Signaling Activation

**Figure 3:**
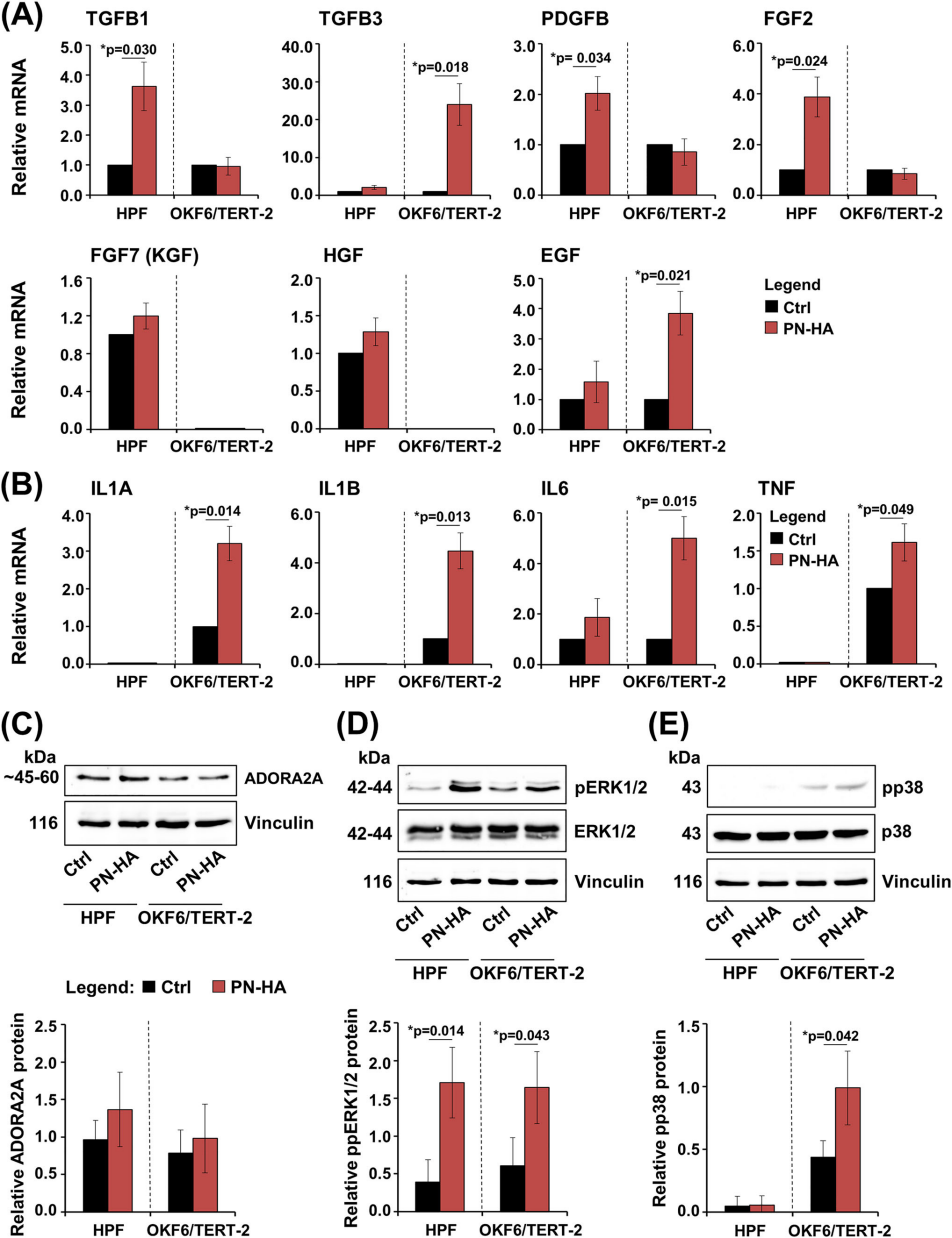
PN–HA modulates distinct growth factors, cytokines, and signaling pathways in oral fibroblasts and epithelial cells. (A, B) Effect of PN–HA on TGFB1, TGFB3, PDGFB, FGF2, FGF7 (also known as KGF), HGF, and EGF (A), and on IL1A, IL1B, IL6, and TNF (B) mRNA levels in HPFs and OKF6/TERT‐2. Cells were treated with PN–HA for 72 h before total RNA was extracted and analyzed by qRT‐PCR. Values normalized to GAPDH are expressed relative to the values of untreated control (Ctrl) cells. Data represent means ± SD from three independent experiments performed with three different cell donors for the HPFs. Significant differences to the respective control, *p < 0.05. (C–E) Immunoblot analyses of ADORA2A (C), phospho‐ERK1/2 (pERK1/2) (D), and phospho‐p38 (pp38) (E) proteins in whole‐cell lysates of PN–HA‐treated HPF and OKF6/TERT‐2. Bar charts represent densitometric quantifications of the immunoblots. ADORA2A levels are normalized to vinculin loading control; pERK1/2 and pp38 levels are normalized to the respective total proteins. Data represent means ± SD from three independent experiments performed with three different cell donors for the HPFs. Significant differences to the respective control (Ctrl) cells, *p < 0.05.

To explore whether PN–HA influences fibroblast proliferation and the migration of both fibroblasts and epithelial cells by modulating key wound healing factors, we analyzed the expression of several relevant growth factors by qRT‐PCR. In HPFs, PN–HA treatment significantly increased mRNA levels of TGFB1, PDGFB, and FGF2 compared to untreated controls (Figure 3A), whereas TGFB3, FGF7 (also known as KGF), HGF, and EGF remained unchanged. Interestingly, in OKF6/TERT‐2 cells, PN–HA selectively upregulated TGFB3, a marker associated with scarless fetal wound healing (Lichtman et al. 2016), and EGF, which is known to promote epithelial differentiation (Gibbs et al. 2000).

We also assessed the impact of PN–HA on inflammatory cytokine expression. With the exception of IL6, transcripts of IL1A, IL1B, and TNF were undetectable in HPFs (Figure 3B). However, PN–HA treatment significantly induced all four pro‐inflammatory cytokines in OKF6/TERT‐2 by 1.6‐ to 5‐fold compared to control, indicating a potential immunomodulatory effect in epithelial cells.

To investigate underlying mechanisms, we examined the expression of the ADORA2A receptor, possibly activated by the PN component, and the phosphorylation states of two mitogen‐activated protein kinases (MAPKs), ERK1/2 and p38, which may be influenced by the HA component. Immunoblotting showed a mild, nonsignificant increase in ADORA2A protein levels in both cell types (Figure 3C). However, ERK1/2 phosphorylation was significantly increased in both HPFs and OKF6/TERT‐2 following PN–HA treatment (*p* < 0.05; Figure 3D), while p38 activation was significantly enhanced only in OKF6/TERT‐2 (*p* < 0.05; Figure 3E).

In summary, PN–HA appears to differentially regulate growth factor and cytokine expression in oral fibroblasts and epithelial cells, which aligns with the observed effects on cellular proliferation and migration. PN–HA also promotes pro‐inflammatory cytokine expression and activates p38 signaling in OKF6/TERT‐2 cells, whereas ERK1/2 signaling is activated in both cell types.

### 3.4 PN–HA Significantly Increases the Expression of Intermediate and Late Keratinocyte Differentiation Markers in Oral Epithelial Cells

**Figure 4:**
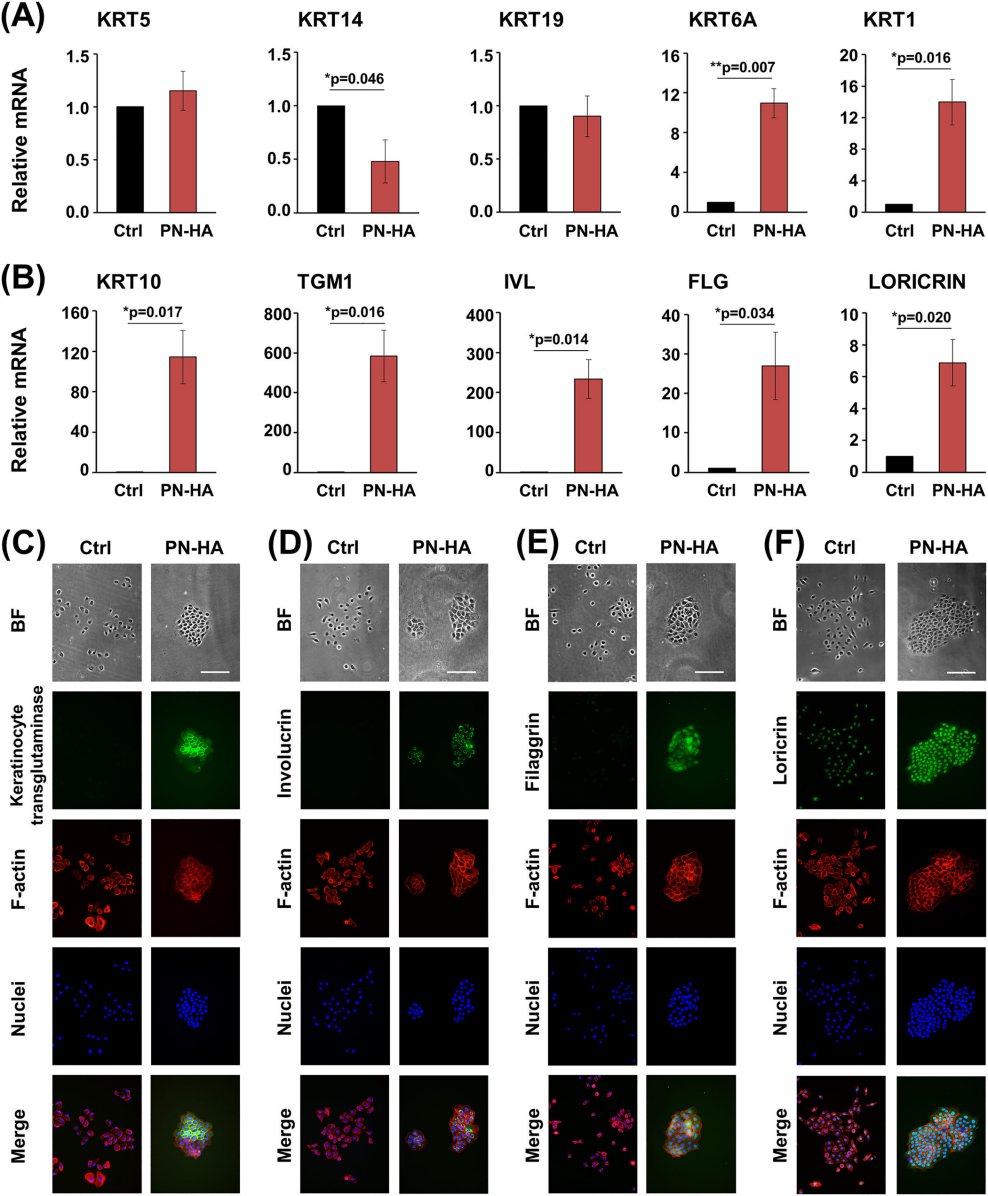
PN–HA significantly increases the expression of intermediate and late keratinocyte differentiation markers in oral epithelial cells. (A, B) mRNA expression levels of KRT5, KRT14, KRT19, KRT6A, and KRT1 (A), and KRT10, TGM1, IVL, FLG, and LORICRIN (B), were measured by qRT‐PCR in OKF6/TERT‐2 cells after 72 h of PN–HA treatment. Values normalized to GAPDH are expressed relative to the values of untreated control (Ctrl) cells. Means ± SD from three independent experiments and significant differences to control, *p < 0.05 are shown. (C–F) Control or PN–HA‐treated OKF6/TERT‐2 was cultured for 72 h before immunofluorescent staining. Keratinocyte transglutaminase (C; green), involucrin (D; green), filaggrin (E; green), loricrin (F; green), F‐actin (red), and nuclei (blue) were visualized on fixed and permeabilized cells. Scale bar, 500 μm. BF, bright field.

We next investigated the ability of PN–HA to induce differentiation of OKF6/TERT‐2 cells by analyzing the expression of various keratinocyte differentiation markers at both the mRNA and protein levels (Figure 4). qRT‐PCR analyses revealed that genes encoding keratins characterizing basal proliferative cells in the oral epithelium were either unaffected by PN–HA, such as KRT5 and KRT19, or significantly downregulated, such as KRT14, compared to control cells (Figure 4A). In contrast, KRT6A and KRT1, which encode keratins characteristic of the suprabasal layer of oral epithelium, were significantly upregulated by 11‐ and 14‐fold, respectively, compared to their expression in control cells. Finally, KRT10, TGM1, IVL, FLG, and LORICRIN genes encoding the late differentiation markers keratin 10, keratinocyte transglutaminase, involucrin, filaggrin, and loricrin, respectively, were all significantly (*p* < 0.05) upregulated in PN–HA‐treated OKF6/TERT‐2 cells (Figure 4B). These transcriptional changes were further confirmed at the protein level. Compared to the dispersed morphology of untreated control cells, PN–HA treatment induced the formation of tightly packed epithelial colonies that expressed keratinocyte transglutaminase, involucrin, filaggrin, and loricrin proteins as shown by immunofluorescence (Figure 4C–F).

These data strongly suggest that PN–HA may have the potential to promote keratinization.

### 3.5 PN–HA Significantly Stimulates Oral Epithelial Cell Proliferation in a Coculture Model With Oral Fibroblasts

**Figure 5:**
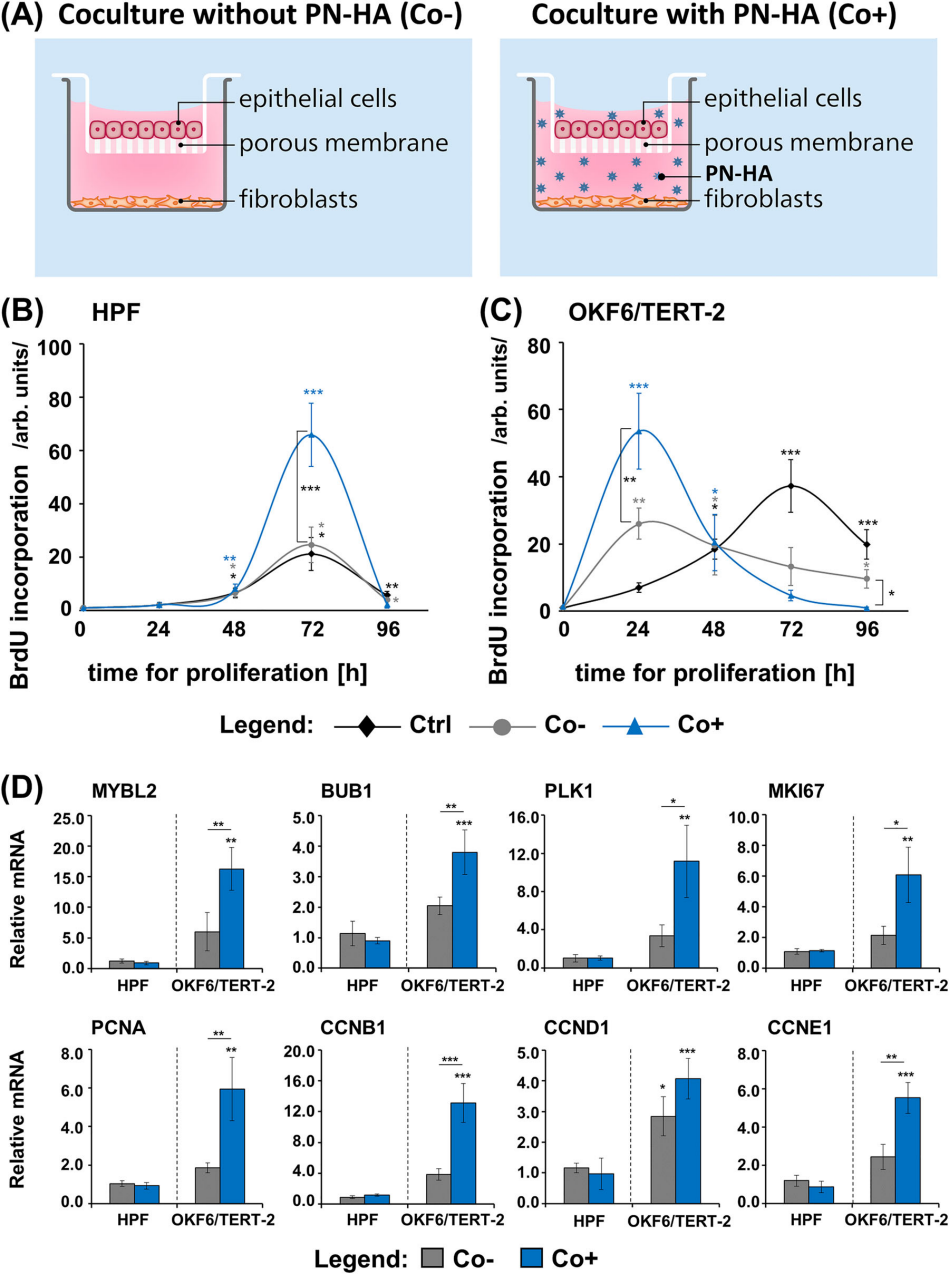
PN–HA promotes oral epithelial cell proliferation in a coculture model with oral fibroblasts. (A) Schematic representation of the indirect coculture model using transwell inserts that allow paracrine signaling through a porous membrane (0.4 µm‐pore size) between HPFs (in the lower chamber) and OKF6/TERT‐2 (in the insert). Two experimental conditions are shown: coculture (1) without PN‐HA (Co−) and (2) with PN–HA (Co+). (B, C) Proliferation of HPFs and OKF6/TERT‐2 in monoculture without PN–HA (Ctrl), Co−, and Co+ conditions was assessed by BrdU incorporation at 24, 48, 72, and 96 h. Data represent means ± SD from three independent experiments performed with three different cell donors for the HPFs. Significant differences to Ctrl at the time point 0 unless otherwise indicated, ***p < 0.001, **p < 0.01, and *p < 0.05. (D) Expression of proliferation‐related genes in HPFs and OKF6/TERT‐2 after 24 h in the indicated conditions, analyzed by qRT‐PCR. Values normalized to GAPDH are expressed relative to the values of Ctrl (set to 1; omitted from graphs for clarity). Data represent means ± SD from three independent experiments performed with three different cell donors for the HPFs. Significant differences to Ctrl unless otherwise indicated, ***p < 0.001, **p < 0.01, and *p < 0.05.

To better simulate the in vivo wound environment where oral fibroblasts and epithelial cells coexist, we established an indirect coculture model using transwells (Figure 5A). HPFs cocultured with OKF6/TERT‐2 cells in the absence of PN–HA (Co−) showed a proliferation pattern similar to that of monocultured untreated HPFs (Ctrl), with a peak at 72 h (Figure 5B). In contrast, coculture in the presence of PN–HA (Co+) significantly (*p* < 0.001) enhanced HPF proliferation by 2.7‐fold.

Monocultured untreated OKF6/TERT‐2 (Ctrl) reached confluence at 72 h (Figure 5C). However, when cocultured with HPFs and treated with PN–HA (Co+), their proliferation increased markedly (*p* < 0.01) during the first 24 h, by 2.1‐fold compared to cocultures without PN‐HA (Co−). Consistent with these results, qRT‐PCR analyses at 24 h showed a significant upregulation of all tested proliferation‐associated genes in PN–HA‐treated cocultured OKF6/TERT‐2 cells, compared to both untreated mono‐ and coculture conditions (Figure 5D).

In summary, PN–HA significantly enhances oral epithelial cell proliferation only in the presence of fibroblasts, highlighting a fibroblast‐mediated mechanism through which PN–HA exerts its pro‐proliferative effect on epithelial cells.

### 3.6 PN–HA Triggers Expression of the Key Epithelial Modulators FGF7 and HGF in Oral Fibroblasts Cocultured With Epithelial Cells

**Figure 6:**
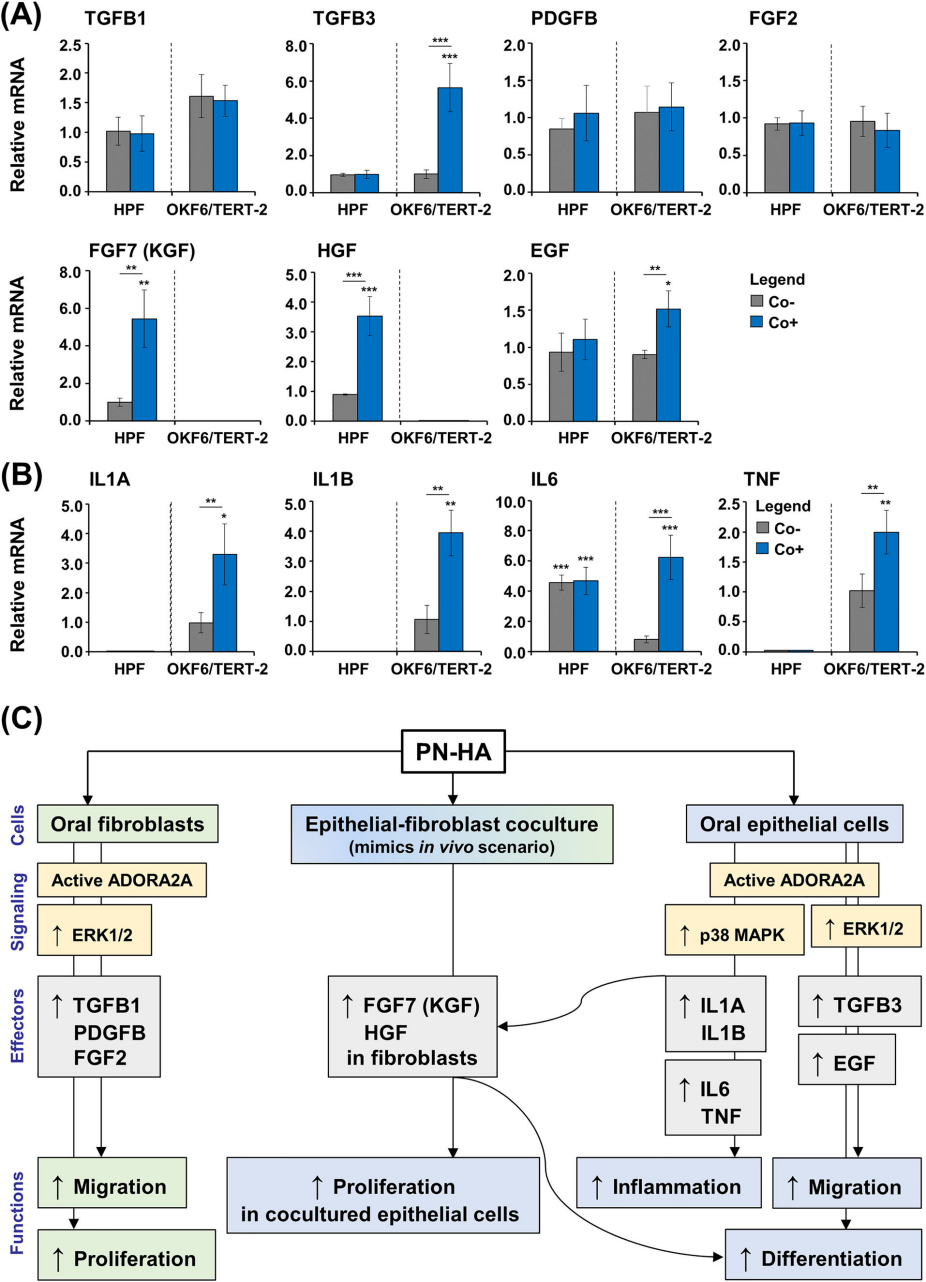
PN–HA induces expression of the epithelial modulators FGF7 and HGF in cocultured fibroblasts. (A, B) Gene expression analysis of wound healing–related growth factors (A) and pro‐inflammatory cytokines (B) in HPFs and OKF6/TERT‐2 cultured for 24 h either in monoculture without PN–HA (Ctrl), coculture without PN–HA (Co−), or coculture with PN–HA (Co+). Total RNA was extracted and analyzed by qRT‐PCR. Values normalized to GAPDH are expressed relative to the values of Ctrl (set to 1; omitted from graphs for clarity). Data represent means ± SD from three independent experiments performed with three different cell donors for the HPFs. Significant differences to Ctrl unless otherwise indicated, ***p < 0.001, **p < 0.01, and *p < 0.05. (C) Schematic overview summarizing the proposed mechanism of action of PN–HA in oral fibroblasts and epithelial cells under mono‐ and coculture conditions. The diagram illustrates the ADORA2A‐associated signaling, stimulation of ERK1/2 and p38 MAPK signaling pathways, upregulation of key effector molecules, and the resulting cell‐ and context‐specific functional outcomes.

We next examined the expression of wound healing‐related growth factors and pro‐inflammatory cytokines in HPFs and OKF6/TERT‐2 after 24 h of coculture, in the absence or presence of PN–HA. At this early time point, neither coculture alone nor PN–HA treatment affected the expression of TGFB1, PDGF, or FGF transcripts in either cell type (Figure 6A). However, in cocultured OKF6/TERT‐2, PN–HA significantly (*p* < 0.05) upregulated TGFB3 and EGF expression above the expression levels detected in untreated mono‐ and cocultures. This was accompanied by a significant increase in IL1A, IL1B, IL6, and TNF expression (*p* < 0.05; Figure 6B). Notably, IL6 mRNA was also significantly (*p* < 0.001) elevated in cocultured HPFs, independent of PN–HA, indicating a coculture‐specific effect.

Importantly, compared to untreated mono‐ and cocultures, in PN‐HA‐treated cocultured HPFs, the expression of two prominent regulators of epithelial cell function (Li et al. 2013; Li et al. 2005), namely FGF7 and HGF encoding the fibroblast growth factor‐7 (also known as keratinocyte growth factor) and the hepatocyte growth factor, respectively, were significantly elevated (*p* < 0.01; Figure 6A). In parallel, PN–HA‐treated OKF6/TERT‐2 cells in coculture showed increased expression of early (KRT5) and late (KRT10, TGM1, IVL, FLG) differentiation markers compared to the control conditions (Figure S1). Based on a well‐established regulatory loop in fibroblast–epithelial cocultures (Werner et al. 2007), the PN–HA‐induced upregulation of IL1A and IL1B in OKF6/TERT‐2 cells likely stimulates the expression of FGF7 and HGF in cocultured HPFs, suggesting a mechanism that could underlie the observed pro‐proliferative and pro‐differentiation effects of PN–HA on epithelial cells under coculture conditions.

## 4 Discussion

During its proliferative phase, oral wound healing is primarily governed by the invasion, proliferation, and differentiation of fibroblasts and epithelial cells (Bartold and Ivanovski 2025). The application of bioactive substances that influence the behavior of these two cell types and thereby promote oral soft tissue regeneration is of major clinical interest (Miron et al. 2025; Perussolo et al. 2025). Accordingly, this study aimed to investigate the in vitro effects of a commercially available PN–HA formulation on the behavior and wound healing–associated gene expression of primary HPFs and oral epithelial cells. A schematic summary of our findings is provided in Figure 6C.

We employed two in vitro models: (1) the immortalized OKF6/TERT‐2 epithelial cell line, a well‐characterized system for studying oral epithelial cell behavior (Dickson et al. 2000; Riaz et al. 2023), and (2) primary HPFs, a key cellular constituent of subepithelial CTGs, which are routinely used in surgical treatment of mucogingival recession defects around natural teeth and dental implants. Previous clinical studies demonstrated the efficacy of crosslinked HA in improving outcomes when used in conjunction with CTGs for root coverage procedures (Guldener et al. 2020; Lanzrein et al. 2020; Pilloni et al. 2019). This raised the clinically relevant question of whether the combination of HA with PN, a compound with documented regenerative effects in non‐dental medical contexts (Lampridou et al. 2025; Vanelli et al. 2010), might yield additional therapeutic benefits in regenerative periodontal procedures. To our knowledge, this is the first in vitro study to provide mechanistic insights into the specific responses of palatal fibroblasts and oral epithelial cells to a combined PN–HA formulation developed for dental application, offering a rationale for further investigation of its therapeutic potential in periodontal surgery.

Our data demonstrate that the PN–HA formulation is fully biocompatible and maintains the viability of both cell types. The biocompatibility of PN–HA has previously been demonstrated (Colangelo et al. 2023; Jiahong et al. 2024; Kim et al. 2020) and appears to be inherent in the physicochemical characteristics of the two components, PN (Marques et al. 2025) and HA (Overmiller et al. 2022). The HA component of PN–HA acts primarily as a hydrated carrier rather than a structural scaffold, providing viscoelastic support and molecular retention but limited cell adhesion capacity (Collins and Birkinshaw 2013; Highley et al. 2016). The slight but not statistically significant decrease in cell viability observed by us at concentrations of PN–HA above 5 mg/mL is likely related to decreased gas and nutrient diffusion within the increasingly viscous matrix, rather than to specific cytotoxicity of PN or HA. Similar oxygen‐transport and diffusion‐limitation effects in dense HA‐based matrices have previously been reported (Collins and Birkinshaw 2013; Shen et al. 2014). In vivo, a 1‐mm‐thick layer corresponds to approximately 1 µL mm^−2^; thus, the 2.5 mg/mL concentration used in vitro approximates the expected effective concentration of the clinical product after partial dilution by oral fluids and/or potential spreading/leakage. At this concentration, PN–HA preserved the morphology of HPFs, as confirmed by cytoskeletal staining for filamentous actin and the type III intermediate filament protein vimentin. Similarly, PN–HA‐treated OKF6/TERT‐2 cells retained epithelial morphology, as indicated by E‐cadherin staining and cluster formation suggestive of early epithelial differentiation.

Our results demonstrate that PN–HA can exert similar but also distinct and complementary effects on fibroblasts and epithelial cells, depending on the culture context. Consistent with previous studies showing a pro‐migratory effect of PN–HA on human dermal (Heo et al. 2025; Jiahong et al. 2024; Kim et al. 2020), and gingival fibroblasts (Colangelo et al. 2021, 2023; Han et al. 2025), as well as HaCaT dermal keratinocytes (Heo et al. 2025), we observed significantly increased migratory capacity in both oral fibroblasts and epithelial cells, while direct stimulation of proliferation was restricted to HPFs. This was accompanied by the selective upregulation of key wound healing–related genes, such as TGFB1, PDGFB, and FGF2, and the activation of the ERK1/2 signaling pathway in HPFs, all known to support fibroblast survival, proliferation, and migration (Guo et al. 2025; Marcopoulou et al. 2003).

Conversely, PN–HA‐treated OKF6/TERT‐2 cells exhibited prominent activation of the p38 MAPK pathway and upregulation of pro‐inflammatory cytokines, suggesting a possible immunomodulatory effect that may contribute to epithelial remodeling during wound healing (Leoni et al. 2015). Interestingly, Han et al. reported anti‐inflammatory effects of the combined application of PN and HA as single‐component medical devices, however on a different cell type and setup, namely upon exposure of gingival fibroblasts to lipopolysaccharides from *Porphyromonas gingivalis* (Han et al. 2025). Furthermore, PN–HA did not stimulate proliferation in monocultured OKF6/TERT‐2 cells, which contrasts with a previously reported pro‐proliferative effect of PN‐HA on HaCaT cells (Heo et al. 2025). This discrepancy may reflect differences in PN–HA formulation and cellular context used. Notably, in coculture with fibroblasts, PN–HA significantly increased epithelial cell proliferation accompanied by the upregulation of multiple cell cycle–associated genes. This suggests that fibroblast‐derived soluble factors, induced or amplified by PN–HA, mediate epithelial responses, highlighting a paracrine mechanism that mirrors the in vivo wound healing dynamics. In addition to its proliferative effects, PN–HA strongly promoted epithelial cell differentiation, as evidenced by the upregulation of intermediate and late keratinocyte differentiation markers. The gene expression changes were pronounced under PN–HA treatment in monocultures and further enhanced in the coculture setting, where fibroblast‐derived mediators likely contributed to the differentiation cascade. From a clinical perspective, these effects are highly relevant as proper epithelial stratification and barrier formation are critical for restoring mucosal integrity after surgical interventions (Wang et al. 2019). The concurrent induction of TGFB3 and EGF observed in PN–HA‐treated epithelial mono‐ and cocultures suggests a coordinated signaling response that promotes re‐epithelialization while restraining hyperproliferation, a balance known to support scarless healing and organized tissue architecture (Overmiller et al. 2022). Notably, TGF‐β3 is associated with scarless fetal wound healing by modulating ECM remodeling and keratinocyte behavior (Lichtman et al. 2016), while EGF is known to facilitate epithelial migration and differentiation without sustaining long‐term proliferative signaling (Matthay et al. 1993).

The dual action of PN–HA in promoting both epithelial proliferation and differentiation, alongside its stimulatory effects on fibroblast‐derived growth factors such as FGF‐7 and HGF, most probably via the PN–HA‐triggered induction of pro‐inflammatory cytokines (Werner et al. 2007), underlies its therapeutic potential in oral soft tissue regeneration. The fibroblast‐derived FGF‐7 and HGF are well recognized for their roles in epithelial‐mesenchymal crosstalk (Werner et al. 2007), where FGF‐7 supports epithelial cell survival, proliferation, and to a lesser extent differentiation (Vintermyr et al. 2003) while HGF predominantly enhances epithelial proliferation and migration (Li et al. 2013). Their upregulation in cocultured fibroblasts upon PN–HA treatment suggests that PN–HA not only supports epithelial expansion but also drives the maturation process required for functional tissue regeneration.

Importantly, the cellular and molecular responses triggered by PN–HA mirror the biological processes that occur during soft tissue healing after root coverage procedures, mucogingival augmentation, and peri‐implant mucosal healing, where both rapid epithelial closure and connective tissue remodeling are essential for long‐term treatment success. Therefore, the observed PN–HA‐driven effects provide a biological rationale for its adjunctive use in periodontal plastic surgery and other regenerative interventions.

While this study provides novel insights into the cellular and molecular mechanisms by which PN–HA enhances oral soft tissue regeneration, certain limitations should be acknowledged. First, the use of an immortalized epithelial cell line (OKF6/TERT‐2) was chosen to ensure experimental consistency and to reduce the complexity of donor variability, particularly in the coculture system where combinations of primary epithelial and fibroblast cells would otherwise be required. However, this approach may not fully capture the heterogeneity and physiological behavior of primary epithelial cells. Second, although the indirect 2D coculture model allowed us to assess paracrine interactions between fibroblasts and epithelial cells, it lacks the structural and functional complexity of in vivo tissue. More advanced 3D organotypic models, which more closely mimic the native architecture of the oral mucosa, could serve as a valuable platform for further validation of our findings. In vivo studies using clinically relevant animal models are also needed to validate the translational significance of our in vitro results and to assess long‐term outcomes such as tissue integration, vascularization, and immune modulation. Non‐crosslinked, high‐molecular‐weight HA is known to undergo enzymatic cleavage by hyaluronidases and oxidative fragmentation by reactive oxygen species within days (Faivre et al. 2024; Žádníková et al. 2022). PNs of natural origin are likewise rapidly degraded by endogenous nucleases once released from the matrix, typically within hours to days (Marques et al. 2025). Accordingly, the PN–HA formulation can reasonably be expected to display a progressive resorption profile—characterized by initial hydration within hours, followed by gradual enzymatic degradation over approximately one to 4 weeks—although this assumption remains to be confirmed through dedicated in vivo studies. Lastly, evaluating PN–HA in combination with commonly used regenerative materials such as CTGs or biomimetic scaffolds may provide further insight into how this formulation could be effectively integrated into current clinical protocols for periodontal and peri‐implant soft tissue regeneration.

## 5 Conclusions

This study provides novel mechanistic evidence underlying the pro‐regenerative effects of a polynucleotide–hyaluronan formulation on human oral fibroblasts and epithelial cells. As summarized in Figure 6C, our findings delineate a downstream signaling cascade initiated by PN–HA, involving: (1) ADORA2A receptor‐associated signaling, (2) stimulation of two MAPK signaling pathways, ERK1/2 and p38, (3) upregulation of key effector molecules, and (4) consequent, measurable alterations in cell behavior, as demonstrated by functional assays. These mechanistic insights, combined with the observed cell‐ and context‐specific promotion of viability, migration, proliferation, and differentiation, support the potential of PN–HA as a bioactive adjunct for enhancing oral soft tissue regeneration and keratinization in periodontal reconstructive applications.

## Author Contributions

Maria B. Asparuhova and Anton Sculean contributed to the conception and design of the study. Ina Mladenova, Xiaoqing Song, and Cristina Nica were involved in conducting the experiments. Ina Mladenova, Xiaoqing Song, and Maria B. Asparuhova were involved in data analysis and interpretation. Maria B. Asparuhova and Ina Mladenova drafted the manuscript. All authors revised the manuscript critically, and gave final approval of the version to be published.

## Ethics Statement

Approval No. 2018‐00661 was granted by the Swiss Ethics Committees on research involving humans. Written informed consent was obtained from the patients.

## Conflicts of Interest

The author declares no conflicts of interest.

## Supporting information


**Table S1:** Primer sequences.
**Figure S1:** PN–HA significantly increases the expression of intermediate and late keratinocyte differentiation markers in oral epithelial cells cocultured with fibroblasts.
**Figure S2:** Uncropped images of Western blots.

## Data Availability

All data generated and analyzed during this study are included in this article and its Supporting Information.
