# Supplementary material for: In Vitro Evidence for Oral Soft Tissue Regenerative Effects of a Polynucleotide–Hyaluronan Formulation
Source: Clin Exp Dent Res. 2025 Nov 18;11(6):e70253. doi: 10.1002/cre2.70253 (PMC12626280; doi:10.1002/cre2.70253)
Supplement: Supplementary file 1 — Table S1: Primer sequences. Figure S1: PN–HA significantly increases the expression of intermediate and late keratinocyte differentiation markers in oral epithelial cells cocultured with fibroblasts. Figure S2: Uncropped images of Western blots. [file CRE2-11-e70253-s001.pdf]

Supporting Information for:

In vitro evidence for oral soft tissue regenerative effects of a polynucleotide–hyaluronan formulation

Ina Mladenova[1], Xiaoqing Song[1], Cristina Nica[1], Anton Sculean[1] and Maria B. Asparuhova[1*]

[1]Laboratory of Oral Cell Biology, Department of Periodontology, School of Dental Medicine, University of Bern, Freiburgstrasse 3, 3010 Bern, Switzerland

***Corresponding author:** Maria B. Asparuhova
Laboratory of Oral Cell Biology
Department of Periodontology
School of Dental Medicine, University of Bern
Freiburgstrasse 3, CH-3010 Bern, Switzerland
Tel: +41 31 684 05 97
Email: mariya.asparuhova@unibe.ch

Content:

Table S1. Primer sequences.

Figure S1. PN-HA significantly increases the expression of intermediate and late keratinocyte differentiation markers in oral epithelial cells cocultured with fibroblasts.

Figure S2. Uncropped images of Western blots.

Table S1 | Primer Sequences.

Gene symbol	Gene bank accession number	Primer pair (fwd/rev)
MYBL2	NM_002466.4	5′-GTCAAATGGACCCATGAGGA-3'<br>5′-GTCAGTGCGGTTAGGGAAGT-3'
BUB1	NM_004336.5	5′-GGAGAACGCTCTGTCAGCA-3'<br>5′-TCCAAAAACTCTTCAGCATGAG-3'
PLK1	NM_005030.6	5′-AACCGAGTTATTCATCGAGACC-3'<br>5′-TTGGTTGCCAGTCCAAAATC-3'
MKI67	NM_002417.5	5′-GAGGTGTGCAGAAAATCCAAA-3'<br>5′-CTGTCCCTATGACTTCTGGTTGT-3'
PCNA	NM_002592	5'-ACACTAAGGGCCGAAGATAACG-3'<br>5'-ACAGCATCTCCAATATGGCTGA-3'
CCNB1	NM_031966.4	5'-CCTCCGGTGTTCTGCTTC-3'<br>5'-TTCAGCATTAATTTTCGAGTTCC-3'
CCND1	NM_053056.3	5'-GCCGAGAAGCTGTGCATC-3'<br>5'-CCACTTGAGCTTGTTCACCA-3'
CCNE1	NM_001238.4	5'-GGCCAAAATCGACAGGAC-3'<br>5'-GGGTCTGCACAGACTGCAT-3'
TGFB1	NM_000660.6	5'-AACCCACAACGAAATCTATGAC-3'<br>5'-GGAATTGTTGCTGTATTTCTGG-3'
TGFB3	NM_001329938.1	5'-GAGCTCTTCCAGATCCTTCG-3'<br>5'-TTTCTAGACCTAAGTTGGACTCTC-3'
PDGFB	NM_002608.3	5'-ATCACCATGCAGATTATGCG-3'<br>5'-GCTCTATCTTTCTTTGGTCTGC-3'
FGF2	NM_002006.4	5'-ACATCAAGCTACAACTTCAAGC-3'<br>5'-CCGTAACACATTTAGAAGCCAG-3'
FGF7 (KGF)	NM_002009.3	5'-AGTTATGATTACATGGAAGGAGGG-3'<br>5'-CACAATTCCAACTGCCACTG-3'
HGF	NM_001010931.2	5'-GCTATCGGGGTAAAGACCTACA-3'<br>5'-CGTAGCGTACCTCTGGATTGC-3'
EGF	NM_001178130.2	5′-AAGATATACTTTGCCCATACAGCC-3'<br>5′-AGAGATTTCCCTCTGTCTGTCC-3'
IL1A	NM_000575.4	5'-GCATGGATCAATCTGTGTCTC-3'

		5'-GGCTTGATGATTTCTTCCTCTG-3'
IL1B	NM_000576.2	5'-AACAGATGAAGTGCTCCTTCC-3'<br>5'-AAGGTGCTCAGGTCATTCTC-3'
IL6	NM_000600.4	5'-GGATTCAATGAGGAGACTTGC-3'<br>5'-TTCTGCAGGAACTGGATCAG-3'
TNF	NM_000594.3	5'-TCTTCTCCTTCCTGATCGTG-3'<br>5'-GAGGGTTTGCTACAACATGG-3'
KRT5	NM_000424.4	5'-TGCTGAAGAAGGATGTAGATGCT-3'<br>5'-TCTGCATCTGGGACAGCTC-3'
KRT14	NM_000526.5	5'-GGAACAAGATTCTCACAGCC-3'<br>5'-GTTCAACTCTGTCTCATACTTGG-3'
KRT19	NM_002276.5	5'-AAGAACCATGAGGAGGAAATCAG-3'<br>5'-CCCGGTTCAATTCTTCAGTC-3'
KRT6A	NM_005554	5'-AGAGCCTTGTATGATGCAGAG-3'<br>5'-CAGCTCCTCGTACTTGGTC-3'
KRT1	NM_006121.4	5'-GGATTACCGGAACAAGTATGAGG-3'<br>5'-TGCATCTGAGACAACTCTGCT-3'
KRT10	NM_000421.5	5'-ACGAGGAGGAAATGAAAGACCT-3'<br>5'-TTCTGTAGTCAGTTCCTTGCTC-3'
TGM1	NM_000359.3	5'-ATTCTGTCTGGAACTTCCATGTG-3'<br>5'-AGAAGATGCCACTGCTAGTC-3'
IVL	NM_005547	5'-TCCTCCAGTCAATACCCATCAG-3'<br>5'-CAGCAGTCATGTGCTTTTCCT-3'
FLG	NM_002016.2	5'-CTGGACACTCAGGTTCCCAT-3'<br>5'-TTTCGTGTTTGTCTGCTTGC-3'
LORICRIN	NM_000427.3	5'-AGACCCAGCAGAAGCAGGCG-3'<br>5'-AGCAGAACTAGATGCAGCCG-3'
GAPDH*	NM_001256799.2	5′-ATCAAGAAGGTGGTGAAGCAG-3′<br>5′-TCGTTGTCATACCAGGAAATGAG-3′

*reference gene used for normalization in all qPCR analyses

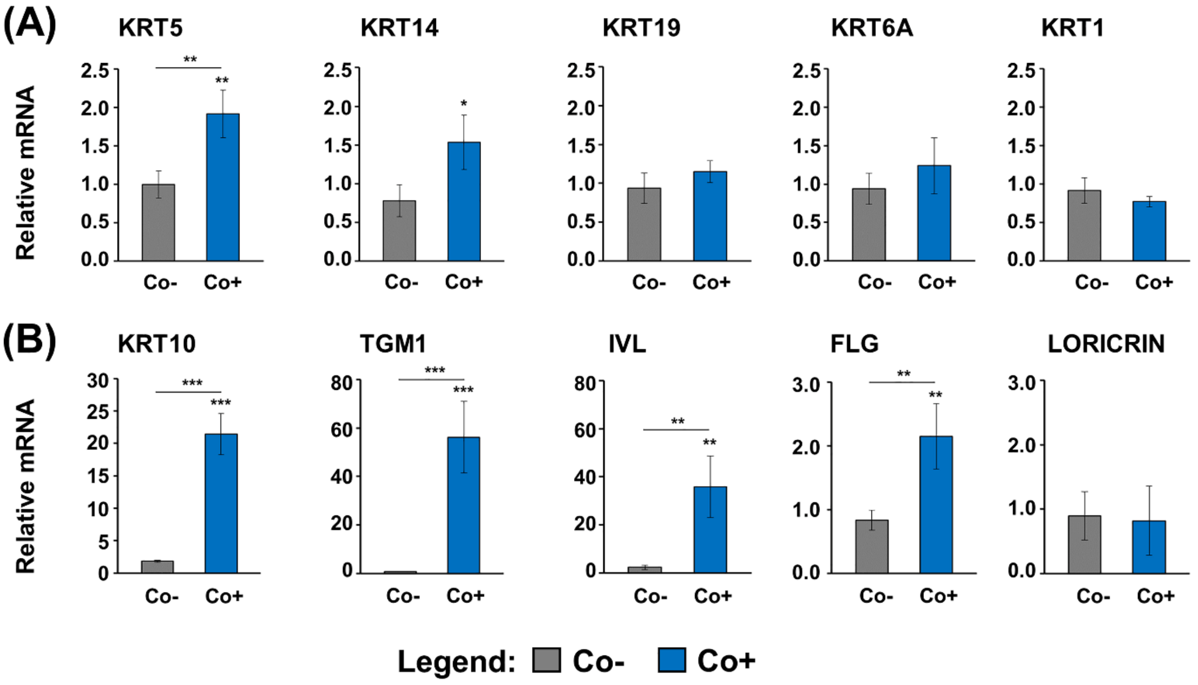

Figure S1 | PN-HA significantly increases the expression of intermediate and late keratinocyte differentiation markers in oral epithelial cells cocultured with fibroblasts. (A, B) mRNA expression levels of KRT5, KRT14, KRT19, KRT6A, and KRT1 (**A**), and of KRT10, TGM1, IVL, FLG, and LORICRIN (**B**), were measured by qRT-PCR in OKF6/TERT-2 cells cocultured with HPFs in the absence (Co-) or in the presence (Co+) of 2.5 mg/ml PN-HA for 24 h. Values normalized to GAPDH are expressed relative to the values of untreated monocultured control cells (set to 1; omitted from the graphs for clarity). Data represent means ± SD from three independent experiments. Significant differences to control unless otherwise indicated, ***$p < 0.001$, **$p < 0.01$, *$p < 0.05$.

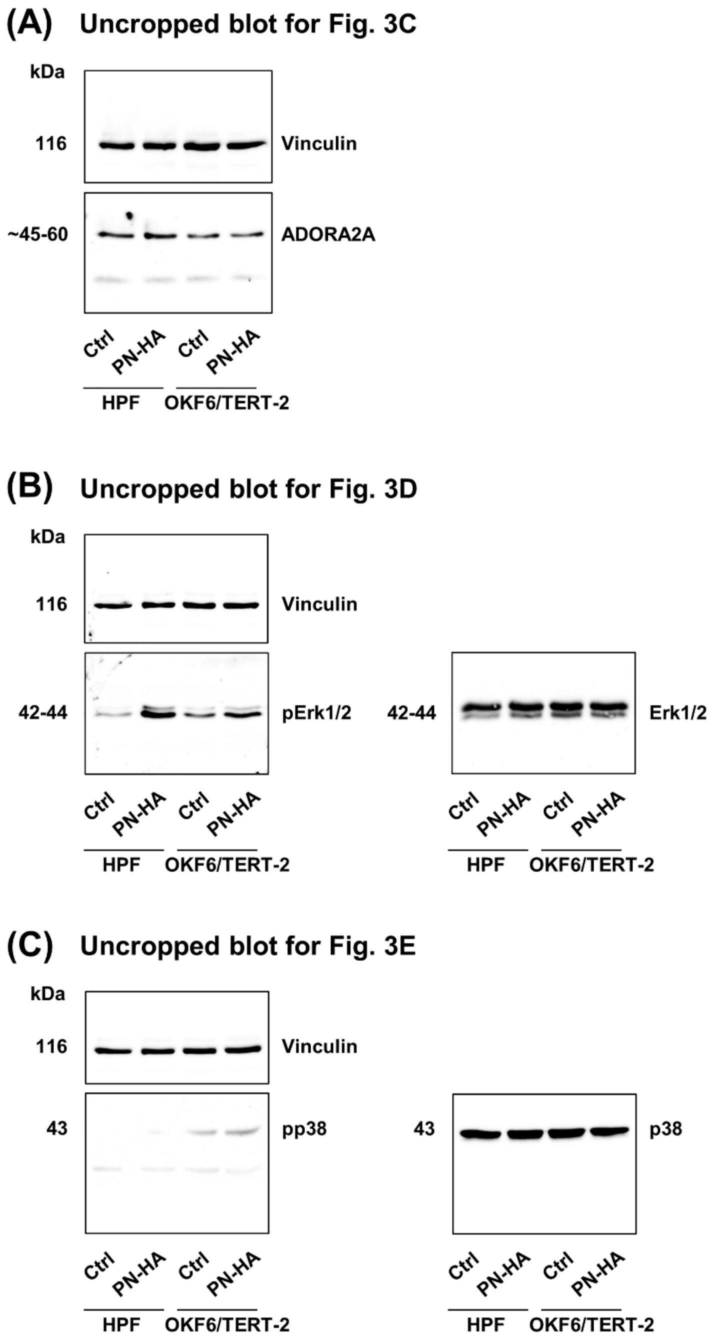

Figure S2 | Uncropped images of Western blots shown in Figure 3C, D, and E. Immunoblot analyses of ADORA2A (**A**), phospho-Erk1/2 (pErk1/2) (**B**), and phospho-p38 (pp38) (**C**) proteins in whole-cell extracts from untreated (Ctrl) or PN-HA-treated HPFs and OKF6/TERT-2 cells. Each of the blots was cut at the level of ~70-80 kDa. The upper part of each blot was developed for the vinculin loading control. The lower part of the blots in (**B**) and (**C**) was first developed for the respective phosphorylated protein. After stripping in buffer containing 62.5 mM Tris-HCl pH 6.8, 100 mM 2-mercaptoetanol, and 2% SDS for 30 min at 50^0C, the lower parts were subsequently blocked in 5% w/v BSA, 1x TBS, 0.1% Tween-20, and developed for the respective total proteins used as internal controls.
